# Suppression of stress induction of the 78-kilodalton glucose regulated protein (GRP78) in cancer by IT-139, an anti-tumor ruthenium small molecule inhibitor

**DOI:** 10.18632/oncotarget.25679

**Published:** 2018-07-03

**Authors:** Suzanne J. Bakewell, Daisy F. Rangel, Dat P. Ha, Jyothi Sethuraman, Richard Crouse, Emma Hadley, Tara L. Costich, Xingliang Zhou, Peter Nichols, Amy S. Lee

**Affiliations:** ^1^ Intezyne Technologies, Inc., Tampa 33612, FL, USA; ^2^ Department of Biochemistry and Molecular Medicine, University of Southern California, Keck School of Medicine, USC Norris Comprehensive Cancer Center, Los Angeles 90089, CA, USA; ^3^ Department of Pathology, University of Southern California, Keck School of Medicine, USC Norris Comprehensive Cancer Center, Los Angeles 90089, CA, USA

**Keywords:** small molecule inhibitor, IT-139, GRP78, ER stress, cancer

## Abstract

In many cancers, combination therapy regimens are successfully improving response and survival rates, but the challenges of toxicity remain. GRP78, the master regulator of the unfolded protein response, is emerging as a target that is upregulated in tumors, specifically following treatment, and one that impacts tumor cell survival and disease recurrence. Here, we show IT-139, an antitumor small molecule inhibitor, suppresses induction of GRP78 from different types of endoplasmic reticulum (ER) stress in a variety of cancer cell lines, including those that have acquired therapeutic resistance, but not in the non-cancer cells being tested. We further determined that IT-139 treatment exacerbates ER stress while at the same time suppresses GRP78 induction at the transcriptional level. Our studies revealed a differential effect of IT-139 on chaperone protein family expression at multiple levels in different cancer cell lines. In xenograft studies, IT-139 decreased BRAF inhibitor upregulation of GRP78 expression in the tumor, while having minimal effect on GRP78 expression in the adjacent normal cells. The preferential decrease in GRP78 levels in tumor cells over normal cells, supported by the manageable safety profile seen in the Phase 1 clinical trial, reinforce the value IT-139 brings to combination therapies as it continues its clinical development.

## INTRODUCTION

An emerging target that plays a critical role in tumor cell survival, tumor progression, and drug resistance is the 78-kDa glucose-regulated protein (GRP78), also known as the immunoglobulin binding protein (BiP) and heat shock protein A5 (HSPA5) [1]. In non-stressed normal cells GRP78 resides in the endoplasmic reticulum (ER) where it regulates the integrity of the ER and the correct folding of newly synthesized proteins [2]. It functions by binding Ca^2+^ to maintain metabolic homeostasis and facilitates the export of misfolded proteins for degradation. GRP78 also forms complexes with ER transmembrane stress sensors: activating transcription factor 6 (ATF6), inositol-requiring enzyme 1 (IRE1), and PKR-like ER kinase (PERK) and maintains them in inactive forms [3]. Under ER stress, misfolded proteins accumulate and GRP78, a hydrophobic protein, binds to the accumulating malfolded proteins to alleviate their aggregation. As GRP78 is titrated away from ATF6, PERK, and IRE1, the unfolded protein response (UPR) is initiated. The UPR, along with other major mechanisms that include translation attenuation, increased expression of ER chaperones, enhanced ER-associated protein degradation and apoptosis, represents an evolutionarily conserved adaptive response that allows cells to overcome proteotoxic stress [4, 5]. Thus, the UPR is an important survival pathway utilized by cancer cells.

GRP78 is expressed in all cell types as an essential chaperone for the synthesis of membrane-bound and secreted proteins processed through the ER [2]. Both young and aged *Grp78* heterozygous mice expressing 50% of wild type GRP78 level are phenotypically normal demonstrating that normal cells can tolerate partial GRP78 down-regulation without adverse effects [6–8]. Nonetheless, in multiple mouse cancer models, *Grp78* heterozygosity potently suppresses tumorigenesis, revealing that cancer progression requires a high level of GRP78, consistent with an elevated level of GRP78 in a wide range of human cancers [1, 7, 9–11]. Therefore, a drug that targets this up-regulation of GRP78, but not the constitutive, basal level of GRP78 could hypothetically present with decreased toxicity effects.

In tumorigenesis, GRP78 induction is mediated not only by intrinsic ER stress, but also as a result of extrinsic factors such as hypoxia and acidosis in the tumor microenvironment. In addition to the requirement of GRP78 for tumor progression and for cancer cell proliferation, tumor-associated endothelial cells also express a high level of GRP78 compared to endothelial cells of normal organs, and GRP78 is required for neoangiogenesis during tumor growth as well as chemoresistance of tumor-associated endothelial cells [12, 13]. GRP78 elevation in tumor cells has been shown to confer resistance to chemotherapeutic drugs, including cisplatin, 5-FU, paclitaxel, docetaxel, sorafenib, bortezomib, etoposide, doxorubicin, temozolomide, vinblastine and camptothecins, as well as anti-hormonal, anti-angiogenesis, chromatin-modifying agents and radiation therapy [1, 14–17]. Decreasing the up-regulation of GRP78 in response to treatment potentially reduces resistance and thereby should increase efficacy of current standard of care therapies.

The various cellular locations of GRP78 correlate with the diverse biological activity of the protein in the cancer cell [18]. ER stress also induces alternative splicing of GRP78 that results in a cytosolic isoform (GRP78va) with a prosurvival function [19]. ER stress also translocates GRP78 from the ER to the mitochondria which is functionally and physically interconnected to the ER [20]. Recently it was discovered that ER stress actively promotes GRP78 to localize to the cell surface [21–23], where it functions as a co-receptor for various ligands [18, 24, 25]. An important function of GRP78 at the cell surface is to serve as an upstream regulator of PI3K-AKT oncogenic signaling, but GRP78 itself is also a downstream target of AKT activation [22, 26, 27]. Suppressing GRP78 up-regulation in response to ER stress also negatively impacts GRP78 relocalization to other sites, thereby also inhibiting these biological pathways controlled by GRP78 outside the ER.

GRP78 is a potent anti-apoptotic protein and can suppress apoptosis by several mechanisms depending on context. GRP78 binds caspase-7 (which is localized to the ER) to prevent its activation, and sequesters BIK to release BCL-2 [28, 29]. GRP78 also binds to mitochondrial and cell surface proteins that are involved in apoptotic pathways [30]. When tumor-initiating cells (TICs) in certain tumors express GRP78 on the cell surface, there is an association with self-renewal and suppression of differentiation and radioresistance, suggesting GRP78 surface expression may be a novel biomarker of TICs [31]. Translating this data into the clinic, the circulating plasma level of GRP78 measured from liquid biopsies may potentially identify patient responders and support personal therapy regimens.

Small molecule agents that interfere with the synthesis, stability or activity of GRP78 in cancer cells can suppress its function at various cellular locations. Blocking the stress induction of GRP78 is particularly attractive since a high level of GRP78 is required for tumorigenesis and is in contrast to normal cells that only need a basal level of GRP78 for cell maintenance. Therefore, the impedance of GRP78 induction under stress conditions is expected to suppress tumor growth, tumor angiogenesis, invasion, metastasis and stem cell survival while sparing unstressed normal cells.

It has recently been shown that combination therapies will be necessary to avoid the emergence of drug resistance in solid tumors [32]. However, the challenge with combination therapy is the increase in drug-drug interaction [33] and limiting patient toxicities [34]. Many preclinical studies will evaluate sensitivity to targeted drugs, but do not evaluate the effect on normal cells, nor the potential for overlapping toxicities in the patient, which are therefore dependent on empirical clinical trials.

IT-139, sodium trans-[tetrachlorobis(1H-indazole) ruthenate(III)], is an intravenously administered small molecule compound that in a US Phase 1 single agent study was well tolerated with modest anti-tumor activity [35]. Side effects were manageable at the maximum tolerated dose of 625 mg/m^2^ and did not include marrow suppression associated with standard cytotoxins. Here, we show that IT-139 is effective in suppressing the stress induction of GRP78 in a wide range of cancer cells via multiple mechanisms but has minimal effect on GRP78 stress induction in the normal human cell lines and primary cells that we tested. In xenograft models, IT-139 alone is capable of suppressing GRP78 expression in tumors but is more effective in combination with chemotherapy. GRP78 level in surrounding normal tissues is not affected. The selective down-regulation of GRP78 in tumors not only provides one mechanistic explanation for the anti-neoplastic activity of this novel compound, but also suggests potential for combination therapy in the clinic with a limited associated increase in toxicity.

## RESULTS

### IT-139 is a ruthenium-containing small molecule antitumor drug capable of suppressing stress induction of GRP78

**Figure 1:**
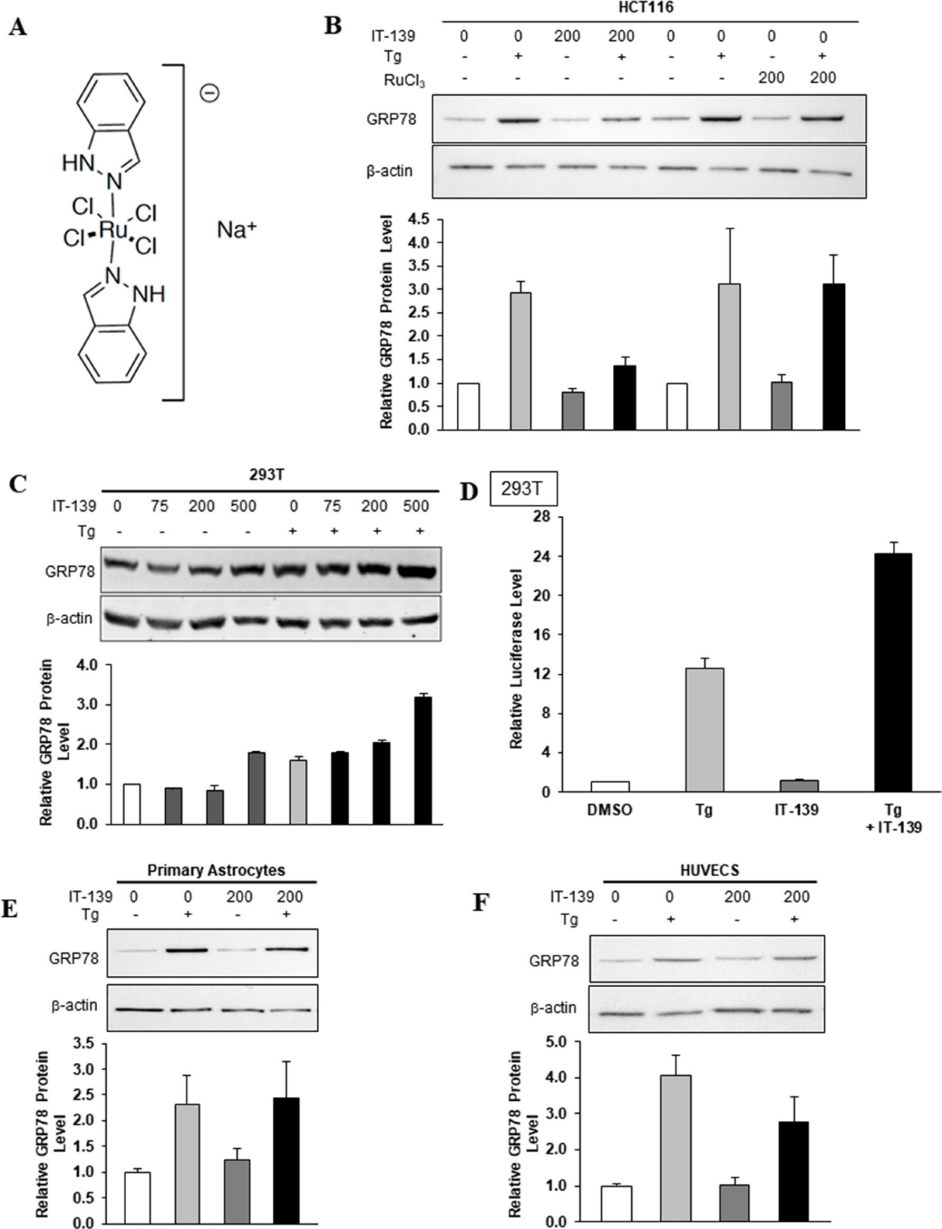
IT-139 molecule and its effects on human cells (**A**) Chemical structure of IT-139: sodium trans-[tetrachlorobis(1H-indazole)ruthenate(III)] (molecular weight 502.15g/mol and formula C_14_H_12_Cl_4_N_4_RuNa). (**B**) Effect of IT-139 and RuCl3 on GRP78 protein in treated HCT116 cells. Relative GRP78 protein levels were measured after 16 hours of treatment by Western blot with β-actin serving as loading control. The band intensities were quantitated and graphed below. Tg: thapsigargin. (**C**) HEK-293T cells were treated with the indicated dosages of IT-139 (0–500 μM) for 16 hours and assayed for GRP78 protein levels. (**D**) HEK-293T stable cell line harboring the -169-luciferase construct was treated with Tg alone or in combination for 16 hours with 200 μM IT-139 as indicated and assayed for luciferase activity. (**E**) Relative GRP78 protein levels in primary astrocytes after 200 μM IT-139 treatment alone or in combination with Tg. (**F**) Relative GRP78 protein levels in HUVECS after 200 μM IT-139 treatment alone or in combination with Tg.

IT-139 (Figure 1A) was selected from a library of ruthenium compounds for its anti-tumor activity in a broad range of human cancer cell lines [36]. The ruthenate anion is susceptible to hydrolysis in aqueous solutions [37], but theoretically is thought to be suppressed in humans by binding to albumin and proteins [38, 39]. To determine if ruthenium is the active metabolite of IT-139 we treated HCT116 colon carcinoma cells with IT-139 and compared the results to the toxicity of ruthenium chloride (RuCl_3_). Cytotoxicity in these studies is defined as the maximal concentration that inhibits the viability of cells by 50% (IC50), or the half-maximal effective concentration (EC50) that induces a response compared to untreated control cells at 72 hrs. The IC50 and EC50 of IT-139 in the majority of cell lines tested are in the micromolar (µM) range (Table 1). We treated HCT116 colon carcinoma cells with RuCl_3_ and compared the IC50 to that of IT-139 treatment. The IC50 of IT-139 in HCT116 cells was 167 μM, but RuCl_3_ showed little toxicity and 50% viability was not reached at 72 hours (not shown).

**Table 1:** IC_50_ and EC_50_ cell viability assay

Cell line	IC50 (µM)		Cell line	EC50 (µM)	
	IT-139	Cisplatin		IT-139	Cisplatin
Capan-1	34.7	2.0	BxPC-3	27.1	2.7
HCT116	167.0	11.5	Capan-1	45.2	10.6
HT-29	15.2	20.3	DU 145	80.8	49.5
A549	148.0	6.6	NCI-H322M	159.0	52.3
A375	130.0	2.6	H1975	33.6	11.4
SKMEL-5	144.0	6.2	LNCaP	17.3	10.3
			MCF-7	37.8	8.0
			MIAPaCa-2	57.4	21.1
			MX-1	104.0	16.0
			N87	3.7	21.3
			PANC-1	30.6	11.1
			PC-3	41.8	18.7
			ZR-75-1	18.1	17.5

To further determine if the ruthenate anion is the active moiety of IT-139, we compared by immunoblot the effect of RuCl_3_ on GRP78 protein levels in HCT116 cells after 16 hours, compared to GRP78 protein levels after 200 µM IT-139 treatment at the same time point (Figure 1B). Thapsigargin (Tg) is a sarco/endoplasmic reticulum Ca^2+^-ATPase (SERCA) inhibitor that induces endoplasmic reticulum (ER) stress by blocking Ca^2+^ reuptake into the ER. 300 nM of Tg treatment for 16 hours increased GRP78 levels 3-fold in HCT116 cells. Tg-induced GRP78 protein levels were reduced by IT-139 treatment to below 1.5-fold compared to control levels, but RuCl_3_ treatment did not affect Tg-induced GRP78 levels, further suggesting that the ruthenate anion is not the active moiety.

### The effect of IT-139 on the stress induction of GRP78 in human cells

GRP78 protein levels are elevated in stressed human cells. To determine whether IT-139 treatment affects stress induction of GRP78 in normal human cells, we utilized the HEK-293T (293T) human embryonic kidney cell line. The 293T cells were treated with IT-139 alone or in the presence of Tg. We observed that in both non-stressed and Tg-stressed cells, IT-139 up to 200 µM showed no or minimal effect on GRP78 protein levels, whereas a stimulatory effect was observed at 500 µM (Figure 1C). Next, we examined the effect of IT-139 on the promoter activity of the *GRP78* gene. For these experiments, we utilized a 293T stable cell line harboring the -169-luciferase construct which contains the three ER stress response elements of the rat *GRP78* promoter fused to a luciferase reporter gene [40]. IT-139 treatment alone at 200 µM showed no effect on the luciferase activity, but in Tg-treated cells, IT-139 further increased the luciferase activity by about 2-fold (Figure 1D).

IT-139 treatment had no effect on GRP78 protein levels in non-stressed primary astrocytes (Figure 1E) or HUVEC primary human endothelial cells (Figure 1F). Combination treatment with IT-139 and Tg in primary astrocytes did not see a change in GRP78 levels over Tg treatment alone (Figure 1E). Interestingly, in contrast to mature endothelial cells in normal organs that exhibit low proliferative rates, HUVECs in culture resemble fast proliferating tumor-associated endothelial cells [13]. We observed about a 1.3-fold decrease in GRP78 levels with 200 µM IT-139 in combination treatment with Tg (Figure 1F).

### IT-139 suppresses stress induction of GRP78 in both androgen-dependent and resistant prostate cancer cell lines and exacerbates ER stress

**Figure 2:**
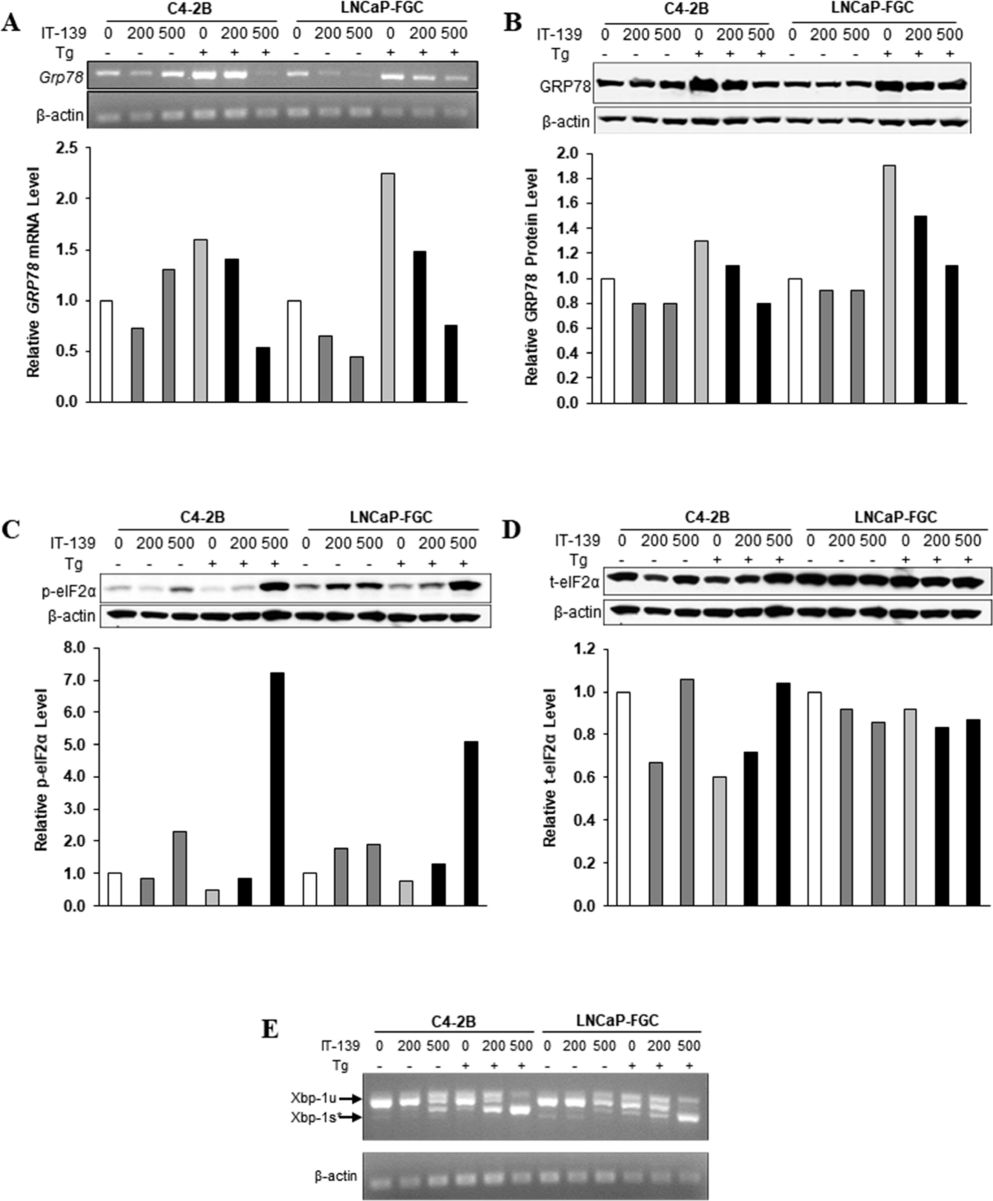
IT-139 specifically suppresses stress induction of GRP78 mRNA and protein (**A**) Prostate cancer cell lines C4-2B and LNCaP-FGC were treated with 200 µM and 500 µM IT-139 alone or in the presence of Tg. *GRP78* mRNA levels were suppressed in both cell lines in stressed and non-stressed conditions. (**B**) Protein levels detected by Western blot showed suppression of GRP78 in stressed conditions in both cell lines. (**C**) Western blots showing protein levels of phosphorylated eIF2α in stressed and non-stressed prostate cells. 500 µM IT-139 results in an increase in eIF2α levels in stressed conditions, and the effect is more sensitive in LNCaP-FGC cells. (**D**) Total eIF2α protein levels are little affected by IT-139. (**E**) Splicing of *XBP-1* mRNA was increased in stressed C4-2B and LNCaP-FGC cells. Tg: thapsigargin. Xbp-1u: unspliced. Xbp-1s: spliced.

To compare the effect of IT-139 on therapeutic resistant human cancer cells, we utilized C4-2B (androgen-independent) and LNCaP-FGC (androgen-dependent) human prostate cancer cell lines. The cells were treated with IT-139 alone or in combination with Tg. The levels of *GRP78* mRNA were detected by RT-PCR (Figure 2A) and the levels of GRP78 protein were detected by Western blot (Figure 2B). Our results showed that IT-139 at 200 and 500 µM was effective to suppress the Tg-induction of GRP78 at both the mRNA and protein level. For non-stressed cells, IT-139 showed suppression of *GRP78* mRNA in both cell lines, but not at the level of GRP78 protein, which is a stable protein with a long half life. A mild elevation of *GRP78* mRNA in the C4-2B cells at the 500 µM dose suggests induction of ER stress.

At the onset of the UPR, a hallmark is eIF2α phosphorylation to suppress translation. However, eIF2α activation is transient and usually subsides after 8 hr. For both C4-2B and LNCaP-FGC cells, IT-139 (500 μM) caused a 2- and 5 to 7-fold increase in phosphorylation of eIF2α in non-stressed and Tg-stressed cells, respectively (Figure 2C). LNCaP cells were more sensitive as 200 μM elicited some effect already. The activation was detected at 16 hr, suggesting a sustained effect. The drug has little effect on the level of total eIF2α or β-actin, which served as loading control for the Western blots (Figure 2D). The activation of eIF2α correlated with the depletion of GRP78 under these conditions.

Another hallmark of UPR activation is splicing of the *XBP-1* mRNA. IT-139 at 500 μM (16 hr) caused mild *XBP-1* mRNA splicing in non-stressed C4-2B and LNCaP cells. IT-139 at 500 μM caused large increase in *XBP-1* mRNA splicing in Tg-stressed C4-2B and LNCaP prostate cancer cell lines, consistent with GRP78 depletion (Figure 2E). Collectively, these results showed that IT-139 not only does not impair induction of the eIF2α or XBP arm of UPR, it exacerbates them.

### IT-139 can suppress induction of GRP78 by different types of ER stress in multiple human cancer cells

**Figure 3:**
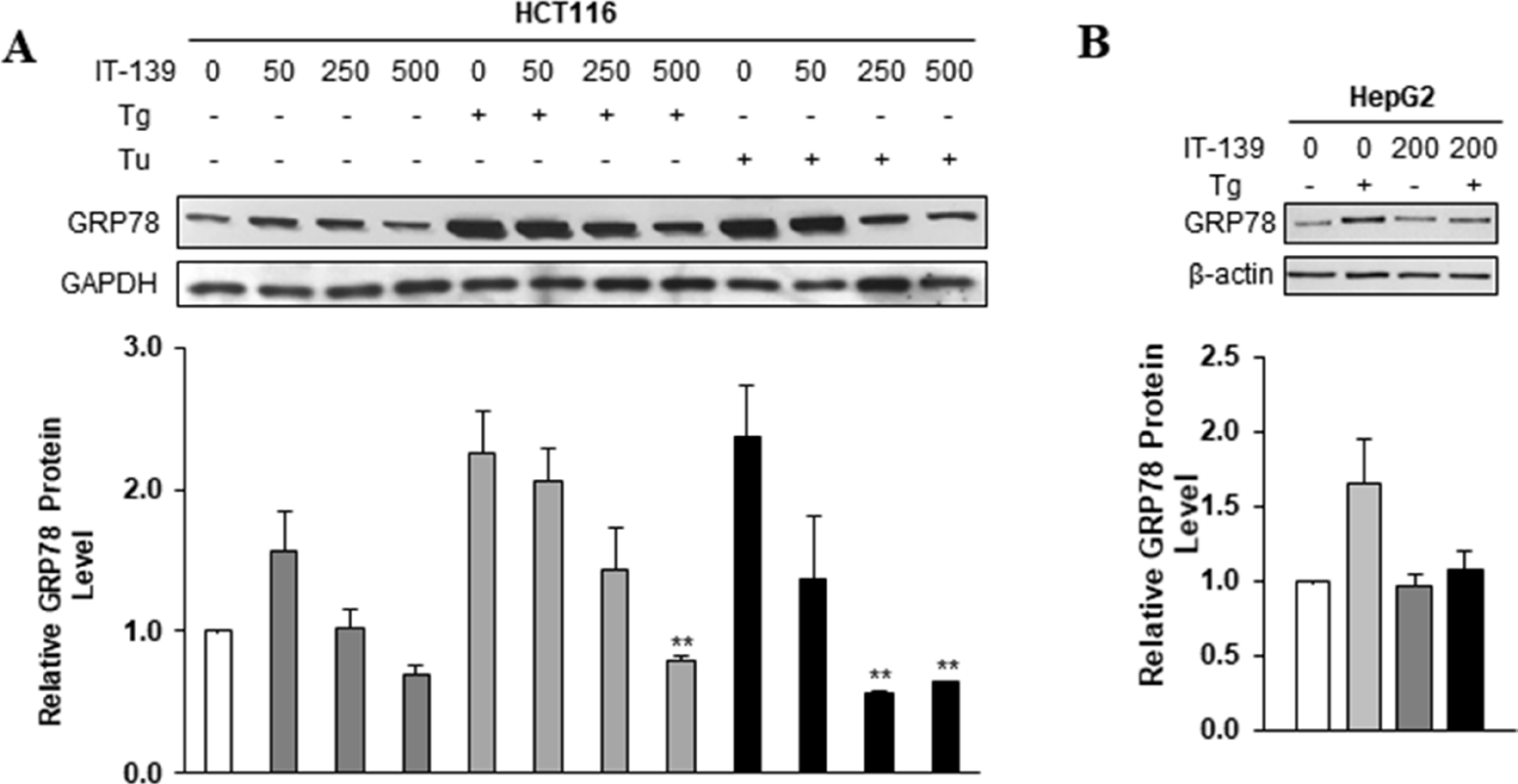
IT-139 suppresses induction of GRP78 by different ER stress inducers (**A**) Colon carcinoma cell line HCT116 was treated with the indicated doses (0–500 µM) of IT-139 alone or in the presence of Tg or Tu. GRP78 protein levels detected by Western blot show suppression of GRP78 under Tg and Tu-induced stress conditions. (**B**) Relative levels of GRP78 were analyzed in hepatocellular carcinoma (HepG2). IT-139 suppressed GRP78 levels under stress conditions. Tg: thapsigargin. Tu: tunicamycin.

To address the lowest effective dose of IT-139 in suppressing GRP78 stress induction and whether this suppression can be observed with another ER stress inducer such as tunicamycin (Tu) which blocks N-linked glycosylation, we treated HCT116 cells with doses of IT-139 ranging from 50 to 500 µM, alone or in combination with Tg and Tu (Figure 3A). Our results showed that for either Tg or Tu induction, 250 µM of IT-139 was sufficient to decrease GRP78 stress upregulation at the protein level. Suppression of Tg-induced GRP78 protein expression at 200 µM IT-139 was also observed in HepG2 (liver) human cancer cells (Figure 3B).

### IT-139 suppresses *GRP78* at the transcriptional level

**Figure 4:**
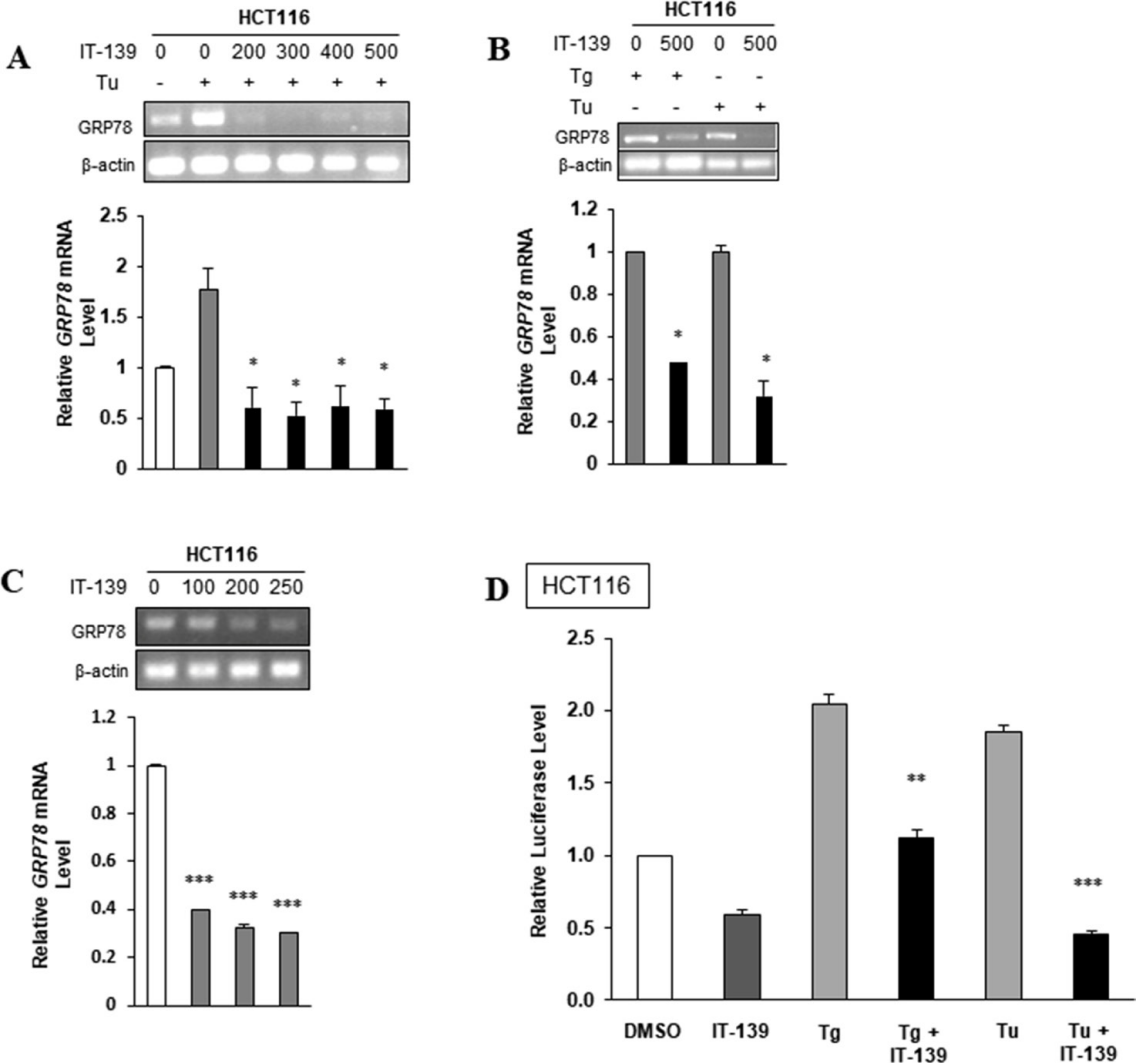
IT-139 suppresses *GRP78* expression at the transcriptional level (**A**) Colon carcinoma cell line HCT116 cells were treated with the indicated dosages of IT-139 (0–500 µM) with or without Tu treatment. (**B**) HCT116 cells were treated with Tg or Tu alone or in the absence or presence of 500 µM IT-139. (**C**) HCT116 cells were treated with the indicated dosage of IT-139 (0–250 µM) for 4 hours and assayed for *GRP78* mRNA levels. (**D**) HCT116 cells were transfected with the -169 luciferase and Renilla luciferase plasmids and treated with Tg, Tu and IT-139, alone or in combination as indicated and assayed for luciferase activity.

To further investigate the effect of IT-139 on *GRP78* transcription, HCT116 cells were treated with Tu in the presence or absence of IT-139 ranging from 200 to 500 µM and assayed for *GRP78* mRNA level by RT-PCR. As expected, Tu treatment resulted in an increase in *GRP78* mRNA levels, but in the presence of IT-139 there was a decrease in *GRP78* mRNA levels and that treatment with 200 µM of IT-139 for 4 hr was sufficient to nearly eliminate Tu induction of *GRP78* mRNA (Figure 4A). Similarly, Tg-induced elevation of *GRP78* mRNA was suppressed by IT-139 (Figure 4B). In these cells, an increasing dosage of IT-139 treatment alone was able to reduce *GRP78* mRNA levels incrementally in non-stressed cells (Figure 4C). The decrease in *GRP78* promoter activity after treatment with 200 µM of IT-139 was also observed in non-stressed and Tg-stressed HCT116 cells, as measured by the luciferase activity in such cells transiently transfected with -169 Luciferase construct which contains the ER stress response elements of the rat *Grp78* promoter driving the expression of the luciferase gene [40] (Figure 4D). The ability of IT-139 to suppress Tg-induced increase in *GRP78* mRNA was confirmed in HT-29 and HepG2 cells (Supplementary Figure 1).

### Differential effect of IT-139 on chaperone protein family expression

To expand our analysis on the effect of IT-139 on gene expression, we analyzed additional human cancer cells. SK-MEL-28 (melanoma) and A549 (lung) were assayed for mRNA and protein level of ER chaperones [GRP78, GRP94, calreticulin (CRT)] and a cytosolic chaperone HSP70. The cells were either non-stressed or treated with Tu and subjected to 0 to 250 µM of IT-139.

**Figure 5:**
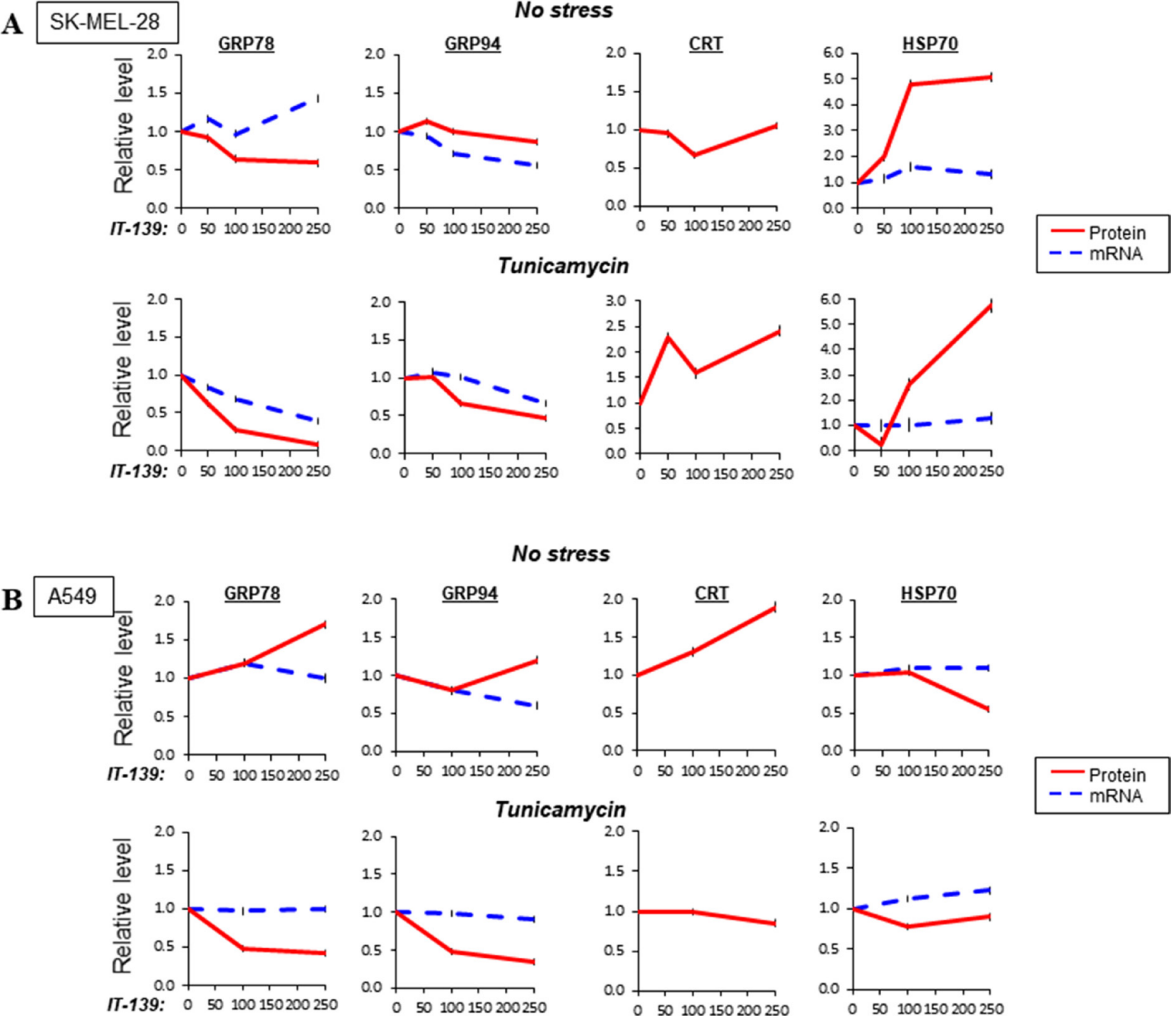
Effect of IT-139 on expression of chaperone protein family (**A**) Relative levels of GRP78, GRP94, CRT and HSP70 in melanoma SK-MEL-28 cells treated with various dosages of IT-139. Protein levels, depicted by a solid line, and mRNA levels, depicted by dashed lines, were measured with or without Tu treatment. (**B**) Same as (A) except lung cancer A549 cells were examined.

In SK-MEL-28 cells, IT-139 mildly increased *GRP78* mRNA at high dose, while moderately suppressed its protein levels in non-stressed cells (Figure 5A, Supplementary Figure 2). It suppressed *GRP94* mRNA, but not protein levels. Tu-induction of *GRP78* mRNA and protein were potently suppressed at 100 µM to 200 µM range. *GRP94* mRNA and protein were moderately suppressed. In contrast, CRT protein and HSP70 protein levels were upregulated, with no effect on *HSP70* mRNA. Therefore, IT-139 suppresses GRP78 and GRP94 stress-induced expression at the transcriptional level, but upregulates HSP70 at the post-transcriptional level in SK-MEL-28 cells.

In lung cancer A549 cells, IT-139 up to 250 µM had no effect on *GRP78* mRNA, but moderately elevated its protein level in non-stressed cells (Figure 5B, Supplementary Figure 3). It suppressed *GRP94* mRNA, but not protein. CRT protein was moderately upregulated and HSP70 mRNA was not affected, but protein was decreased. Tu-induction of *GRP78* and *GRP94* mRNA were not affected at 250 µM, but both GRP78 and GRP94 protein levels were potently suppressed. In contrast, in Tu-stressed cells, CRT protein, *HSP70* mRNA and protein were not affected by IT-139. Therefore, IT-139 suppresses GRP78 and GRP94 stress-induced expression at the post-transcriptional level in A549 cells.

### Over-expression of GRP78 rescues cells from IT-139-induced apoptotic activities

**Figure 6:**
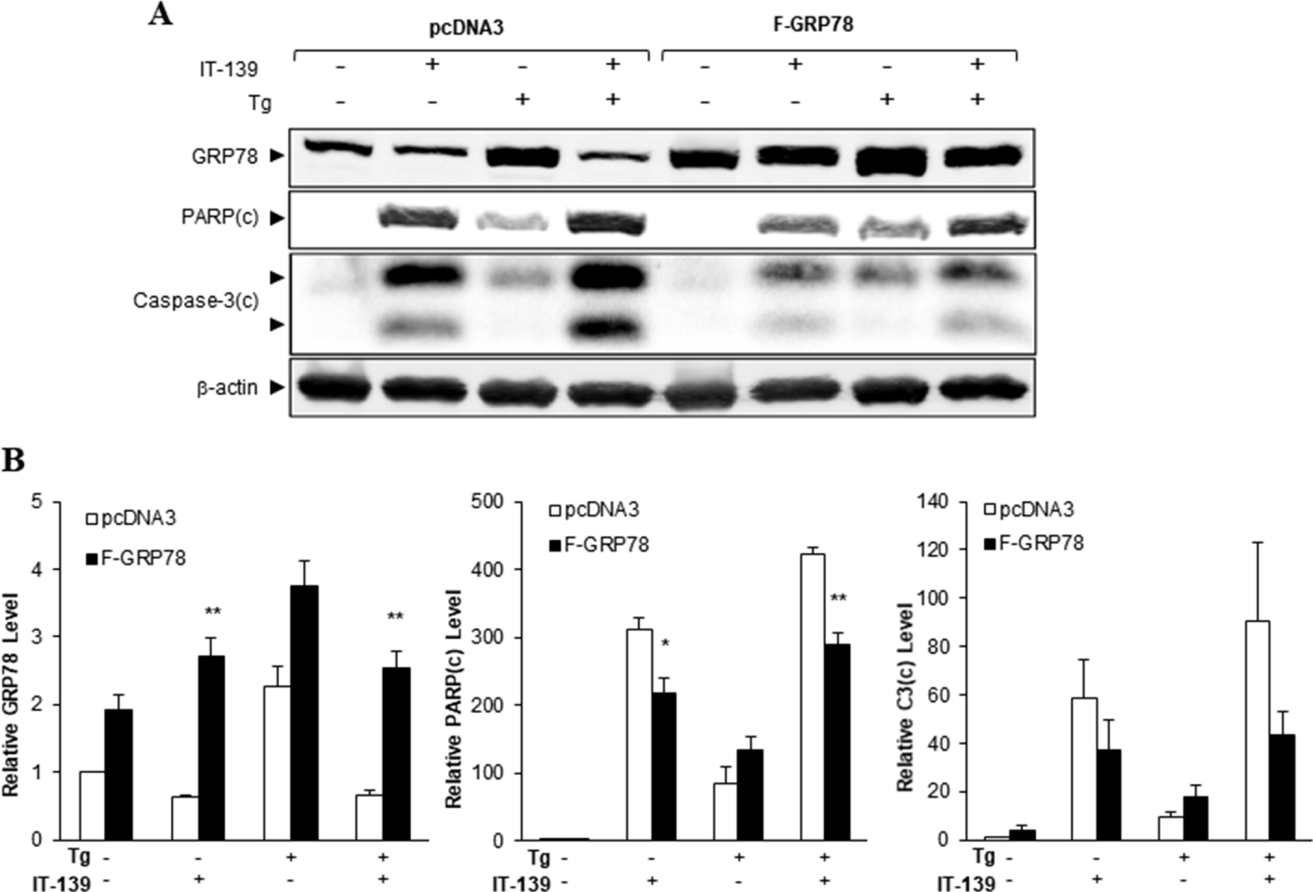
GRP78 over-expression alleviates IT-139-induced apoptotic activities (**A**) HCT116 cells transfected with pcDNA3 empty vector or vector expressing FLAG-GRP78 were treated with IT-139 and Tg as indicated for 24 hr. Whole cell lysates were collected and the indicated proteins were analyzed by Western blot with β-actin as loading control. The cleaved form of PARP and Caspase-3 were denoted as PARP(c) and Caspase-3(c). (**B**) The band intensities for the indicated proteins in (A) were quantified, normalized against β-actin and graphed. The relative levels are shown with standard error.

To determine the importance of GRP78 in the apoptotic action of IT-139, we transiently transfected HCT116 cells with a pcDNA3 vector expressing FLAG-tagged GRP78 (F-GRP78) driven by the CMV promoter or the empty vector. Since transcriptional control of the CMV promoter is unaffected by IT-139, the expression of F-GRP78 in the transfected cells was not suppressed by IT-139 in contrast to endogenous GRP78 (Figure 6A). Over-expression of F-GRP78 was estimated to be about 2-fold over the endogenous level (Figure 6B). Our results showed that this was sufficient to reduce IT-139-induced activation of the apoptotic markers PARP and Caspase-3 by about 50%, under non-stressed and Tg-stressed conditions (Figures 6A and 6B). Collectively, these results indicate that GRP78 suppression contributes significantly to the apoptotic activities of IT-139, which can be rescued through GRP78 over-expression.

### IT-139 synergizes with standard therapy *in vivo*

**Figure 7:**
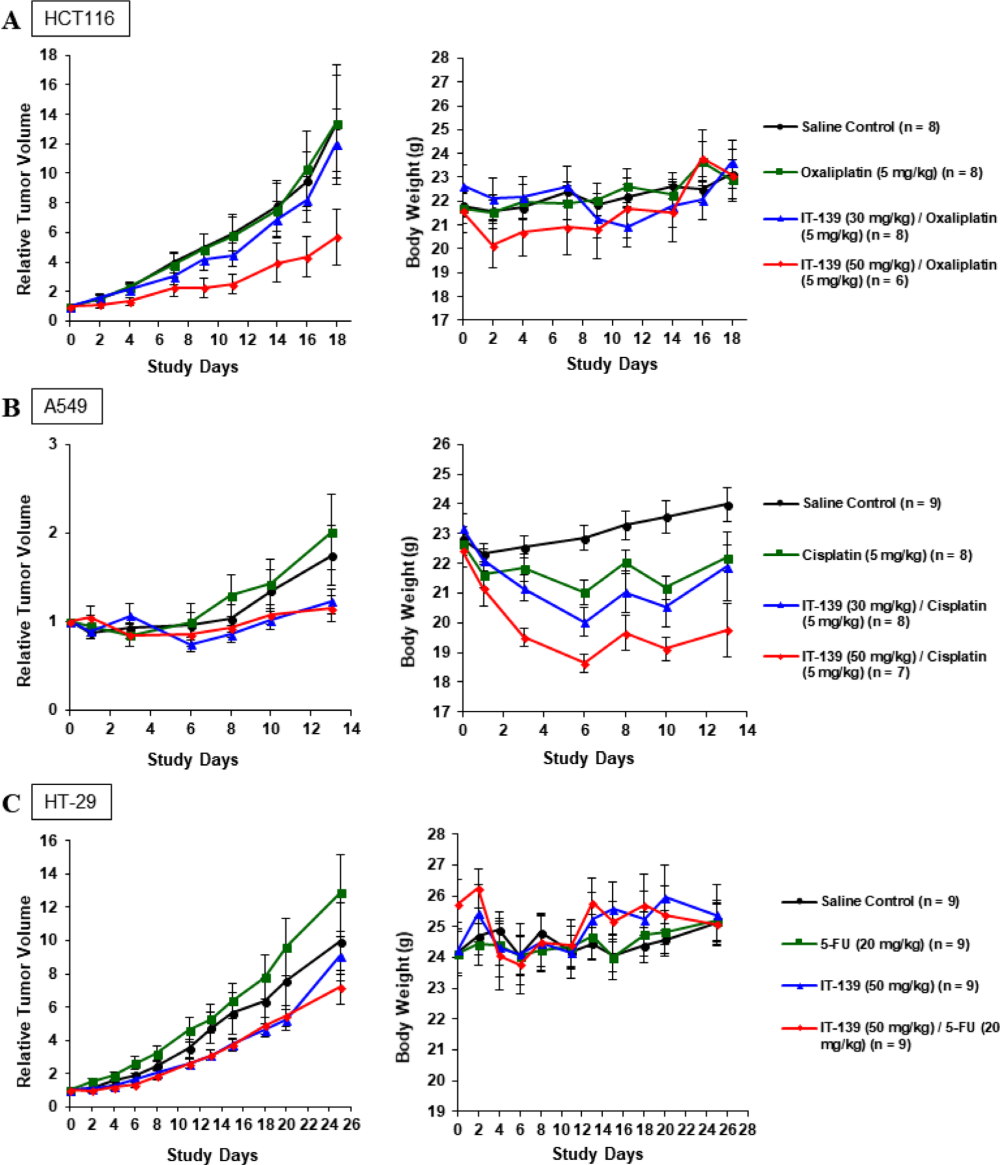
IT-139 treatment *in vivo* (**A**) HCT116 xenograft tumors were treated with saline control, oxaliplatin, or IT-139 in combination with oxaliplatin at 2 doses. Oxaliplatin in combination with IT-139 at 50 mg/kg had significant anti-tumor efficacy, but this group saw the most weight loss. No animal in this group experienced weight loss exceeding 20% of weight at study start. (**B**) A549 xenograft tumors were treated with saline control, cisplatin or IT-139 in combination with cisplatin at 2 doses. Both combination groups saw significant efficacy over the cisplatin treated and control treated groups. The most weight loss was seen in the higher dosed combination group. (**C**) HT-29 xenograft tumor models were treated with saline control, IT-139 alone or in combination with 5-FU at 50 mg/kg. IT-139 was seen to have an effect in both the monotherapy and combination therapy groups. HT-29 cells are 5-FU resistant as observed by the rate of tumor growth in the 5-FU treated group. Although an initial weight loss was seen in the combination group, all treated animals showed normal weight gain in the study. No treated animals in the three xenograft studies experienced weight loss exceeding 20% of weight at study start.

Combination index [41] results (Table 2) show IT-139 synergism with most classes of cancer treatments, suggesting that it can potentially be administered in combination with standard anticancer therapies. We treated three xenograft *in vivo* models (HCT116, A549, and HT-29) with IT-139 at 30 mg/kg every four days, or 50 mg/kg once a week in combination with oxaliplatin, cisplatin or 5-FU respectively (Figures 7A–7C). In all models, IT-139 increased anti-tumor efficacy. Except for the 50 mg/kg dose in the A549 model (Figure 7B), it did not significantly increase toxicity as evaluated by average body weight of the group. Following the A549 cisplatin combination study, the dosing of the chemotherapeutic and IT-139 were staggered by 24 hours. In the HT-29 xenograft model (Figure 7C), we saw a response in anti-tumor growth with IT-139 in combination with 5-FU that was increased at the 50 mg/kg dose. However, the effect on body weight was reduced with the staggered dosing, with a maximum 8% loss compared to the 15% weight loss in the cisplatin group that was dosed in the same bolus as 50 mg/kg IT-139.

**Table 2:** Combination treatment assay

Therapeutic	Cell line	Cancer	Combination index
CDDP	A549	Lung	0.172
Paclitaxel	LNCaP	Prostate	0.219
Doxorubicin	Hep3B	Liver	0.252
Everolimus	MKL-1	NET	0.354
5-FU	HCT-116	Colon	0.36
Oxaliplatin	Lovo	Colon	0.493
Erlotonib	A549	Lung	0.509
Sorafenib	Hep3B	Liver	0.536
Docetaxel	LNCaP	Prostate	0.543
5-FU	Lovo	Colon	0.597
Gemcitabine	A549	Lung	0.647
CDDP	HCT-116	Colon	0.687
Erlotonib	BxPC3	Pancreatic	0.691
Docetaxel	A549	Lung	0.724
CDDP	N87	Gastric	0.757
Sorafenib	A549	Lung	0.846
Gemcitabine	PANC-1	Pancreatic	0.895
Paclitaxel	A549	Lung	1.01
Gemcitabine	Capan-1	Pancreatic	1.02
5-FU	ZR-75-1	Breast	1.05
Docetaxel	N87	Gastric	1.05
Paclitaxel	N87	Gastric	1.1

Combination indices were calculated using the Chou-Talay method. Cells were exposed concomitantly to both drugs for 72 hours and cytotoxicity determined using the MTT assay. The Combination Index (CI) values were calculated using the Compusyn^®^ program. CI values of 0.90–1.10 indicate additive effects. CI values of < 0.90 indicate synergy (the smaller the value, the greater the degree of synergy).

### IT-139 decreases GRP78 expression in treated tumors but not in adjacent normal cells

**Figure 8:**
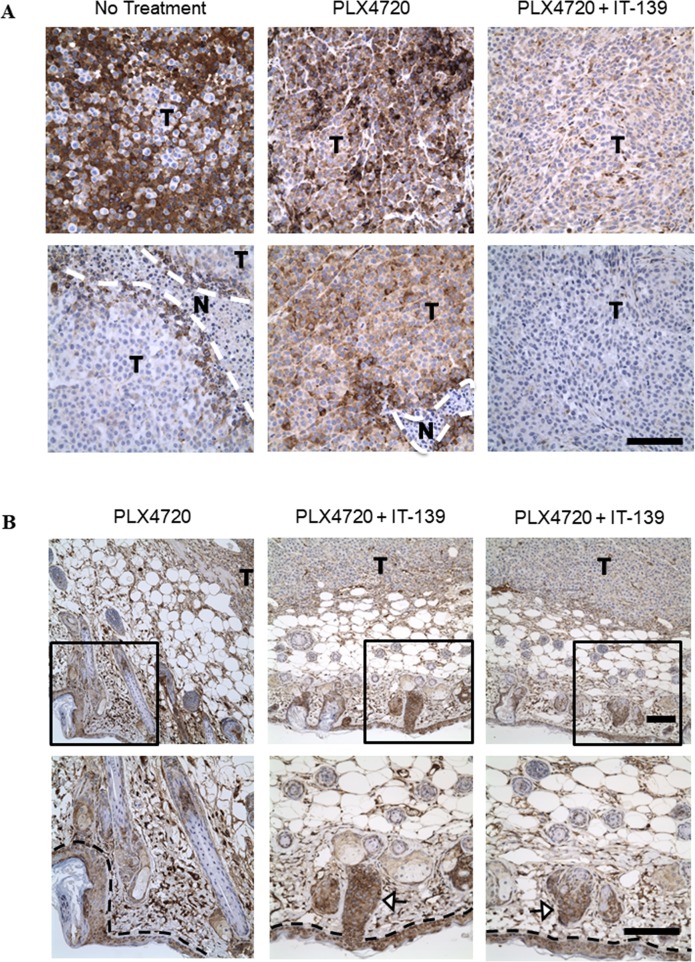
IT-139 preferentially decreases GRP78 expression in tumor cells *in vivo* (**A**) Formalin fixed A375 xenograft tumor sections from mice treated with BRAF inhibitor (PLX4720) alone or in combination with IT-139, or saline as no treatment, were stained for GRP78 expression (brown). T depicts tumor cells and N depicts the necrotic regions. (**B**) A375 tumors treated with either PLX4720 alone or in combination with IT-139 showed GRP78 expression was unaffected by IT-139 in adjacent non-tumor cells as seen in skin (epidermal layer outlined by black dashed line) and hair follicles as signaled by white arrows. Bottom images are a magnification of the boxed area of the image shown on top. Scale bar, 100 μm.

In a BRAF-mutated *in vivo* xenograft study, we treated A375 melanoma tumor-bearing animals with a BRAF mutated inhibitor (PLX4720), IT-139 or both in combination. We harvested the tumors after 15 days, fixed in formalin and stained by immunohistochemistry for GRP78 expression. The A375 tumors from the vehicle (saline) group exhibited heterogeneous GRP78 expression within the tumor region with notably high expression at the necrotic borders (Figure 8A). The PLX4720 treated group had consistent strong GRP78 staining across the tumor sections, whereas the IT-139/PLX4720 treated tumor sections showed greatly reduced GRP78 expression. Thus, PLX4720 appeared to induce higher expression levels of GRP78 that were decreased by combination treatment with IT-139. However, the GRP78 expression in the normal skin cells surrounding the tumor section was not affected by IT-139 treatment (Figure 8B).

## DISCUSSION

GRP78 is highly expressed in a wide variety of cancers and controls multiple steps of tumorigenesis in response to environmental and therapeutic insult. GRP78’s upregulation in tumor cells makes it an attractive target for anti-cancer therapy [1, 14]. Considering that in tumor cells GRP78 can be localized to various cellular locations mediating pro-proliferation and survival functions, agents that inhibit the synthesis, stability or activity of GRP78 will be most effective in suppressing its function at the multiple locations. However, because GRP78 is an essential chaperone, the challenge of anti-GRP78 drug development is to minimize toxicity to normal organs. Heterozygous *Grp78* knockout mouse models in whole body as well as specific organs established that 50% decrease in GRP78 expression has no effect on normal organ function throughout the lifespan, but significantly impedes tumor growth and angiogenesis [1, 8, 13]. This suggests that in adult animals, agents that selectively block the stress induction of GRP78 will primarily affect tumors that require a higher level of GRP78 for proliferation, invasion and therapeutic resistance while sparing normal organs that only need a low basal level of GRP78 for maintenance. These agents should also be able to target tumor-associated cells that fuel cancer growth and require high GRP78 expression for their own growth and survival in the toxic tumor microenvironment.

The interest in GRP78 as a potential target for therapy is rapidly growing because of its pivotal role in ER stress, cell survival, and drug resistance. It has been reported that small molecule inhibitors such as versipelostatin, piericidin A, pyrvinium are able to suppress glucose starvation induction of GRP78, but not always following stress induction by either Tu or Tg [42–44]. This raises the issue whether these agents may actually target mitochondrial function and protein synthesis in general [42]. In this study, we investigated the effect of IT-139 on GRP78 expression *in vitro* and *in vivo*. IT-139 effectively suppresses the stress induction of GRP78 induced by both Tg and Tu, suggesting a distinct mode of action from the small molecule inhibitors described above. The suppression of GRP78 expression by IT-139 in cancer cell lines involves both transcriptional and post-transcriptional mechanisms and appears to be context-dependent. For example, in HCT116 colon cancer cells, IT-139 is able to suppress *GRP78* mRNA in both stressed and non-stressed cells, however, the GRP78 protein level was majorly suppressed only in the stressed cells. One reason IT-139’s IC50 is in the micromolar range *in vitro* could be due to GRP78 level not being decreased sufficiently prior to 72 hours to trigger apoptotic pathways. The high micromolar IC50 level is not necessarily a concern for continued clinical development as the MTD determined in the Phase I Clinical Trial was 625 mg/m^2^ and the pharmacokinetic data values are linear [35].

In non-stressed cells, since there is only low basal synthesis of GRP78, the decrease in *GRP78* mRNA level may not affect the overall GRP78 level within a short time period, depending on the stability of GRP78 in the particular cell line. In the case of HCT116 cells, the GRP78 protein has a half-life of over 40 hours (data not shown), hence the IT-139 suppressive effect was detected at the *GRP78* mRNA level but not at the protein level after 16 hr of IT-139 treatment. In contrast, in stressed cells, *GRP78* is actively being transcribed and translated. Thus, a block in *GRP78* transcription by IT-139, as revealed by *GRP78* promoter-driven luciferase assay, would inhibit stress-induced upregulation of GRP78 protein. Similarly, IT-139 suppresses stress induction of GRP78 at the transcriptional level in LNCAP, C4-2B, HepG2 and SK-MEL-28 cells. However, in A549 lung cancer cells, IT-139 has no effect on *GRP78* mRNA, but moderately elevates its protein level in non-stressed cells and stress induction of *GRP78* mRNA was not affected, but GRP78 protein level was suppressed, implying that IT-139 suppresses GRP78 stress-induced expression at the post-transcriptional level in these cells. Interestingly, the expression of HSP70 protein, which shares 50% amino acid homology with GRP78, is either not affected nor upregulated by IT-139 treatment. This suggests IT-139 is not a general inhibitor of gene transcription, translation or protein stability. While there could be multiple mechanisms mediating the anti-cancer effect of IT-139, we observed that IT-139 could cause changes in cell morphology and vacuolization likely resulting from ER expansion (data not shown). Such changes in ER structure will activate ER stress, and without the benefit of GRP78 induction to buffer the damage, cell viability could be compromised.

Evidence is emerging that small molecules such as OSU-03012 and HA15 that are capable of suppressing GRP78 translation or its ATPase activity could alleviate drug resistance and radioresistance [1, 45, 46]. Recently Gifford *et al.*, showed that GRP78 is significantly higher in gemcitabine-resistant PDAC and that IT-139 treatment *in vivo* increased overall survival with gemcitabine over GEM as monotherapy, and *in vitro* restored sensitivity to cytotoxic drug resistance [47]. The HT-29 cell line is resistant to the chemotherapeutic 5-FU, but we see a response in anti-tumor growth with IT-139 that is increased at the 50 mg/kg dose. In this model, we staggered the dosing by 24 hours, and the gross toxicity and weight loss was not observed as when we dose at the same time, usually in the same bolus. Our hypothesis is that the reported rate of 1.2–1.5 Ru binding to albumin peaks at 1 hour and drops to 1 Ru per albumin at 24 hours [48, 49]. The half-life of circulating 5-FU is short [50], so staggering dosing by 24 hours allows 5-FU to be cleared from albumin before IT-139 binds. Also, the rate of albumin uptake could account for the difference in the effect of IT-139 on GRP78 in highly metabolic cancer cells (including HUVECS) compared to normal cells. Also of note, dosing of IT-139 in rodents is not as high as levels dosed in patients. The maximum tolerated dose in mice is 50 mg/kg that converts to approximately 155 mg/m^2^ in the clinic. This could potentially be due to the reduced molar concentration of albumin in the mice and account for the data reported in the xenograft models. These results require further investigation to determine optimal dosing strategy for future clinical development.

IT-139 shows striking activity in suppressing GRP78 expression in the treated tumors, alone or in combination with standard therapy. To our knowledge, this is the first demonstration that a small molecule is able to suppress GRP78 expression *in vivo*. Importantly, GRP78 levels in the normal skin cells adjacent to the tumor was not affected by the IT-139 treatment, in agreement with minimal effect of IT-139 on GRP78 stress induction in non-cancer cells being tested *in vitro*. The mechanism for this specificity warrants further investigation. In the Phase I dose-escalation open-label study in patients with advanced solid tumors, IT-139 was well tolerated with a manageable toxicity profile. No dose limiting hematologic toxicity was observed and there were no treatment-related deaths in patients treated up to two years with IT-139. Pharmacokinetic (PK) parameters of ruthenium increased proportionally with dose and although there was no evidence of cumulative toxicity, less frequent dosing will be considered for the schedule in future clinical studies. If the stress induction of GRP78 in the tumor is the primary target, then potentially normal cells will be spared and severe adverse events will be reduced. This is especially attractive for combination therapy as increase in toxicity is the rate-limiting step for many current combination dosing regimens.

## MATERIALS AND METHODS

### Cells

HCT116 and HT-29 were cultured in McCoy’s 5a Modified Medium; HUVECs, A549 were cultured in F-12K Medium; Capan-1, A375, HepG2 and 293T stable cell line harboring the -169 luciferase construct (firefly) [40] were cultured in Dulbecco’s Modified Eagle Medium; C4-2B, LNCaP and SK-MEL-28 were cultured in RPMI 1640 Medium. Cell lines for the EC50 and combination assay were obtained from ATCC or the UNC Lineberger Comprehensive Cancer Center and established using standard *in vitro* culture methods and supplier-recommended media and supplements. All media for the above cells were supplemented with 10% (v/v) fetal bovine serum, 1% (v/v) 2 mmol/L L-glutamine and 1% pen strep. Primary astrocytes (HA) were from ScienCell Research Laboratories and cultured in Astrocyte medium (AM, Cat. #1801). All cell lines were cultured in a humidified incubator with 5% CO_2_ and 95% air.

### Protein extraction and immunoblot analysis

Cells were treated with IT-139 alone or in combination with tunicamycin or thapsigargin for 16 hours. Cell lysates were prepared and subjected to 10% or 12% SDS-PAGE and Western blot analysis. The following primary antibodies were used: GRP78 (1:1000, BD Biosciences #610978); β-actin (1:5000, Sigma-Aldrich, #A5316); eIF2α (1:1000, Cell Signaling #2103); phospho- eIF2α (1:1000, Cell Signaling #9721). The secondary antibodies used were as follows: horseradish peroxidase conjugate goat anti-mouse (1:1000, Santa Cruz Biotechnology #sc-2005) and anti-rabbit (1:1000, Santa Cruz Biotechnology #sc-2004).

### Transfection conditions

HCT116 cells were transfected with empty pcDNA3 vector or vector expressing FLAG-GRP78 [21] using BioT reagent (Bioland Scientific) according to manufacturer recommendation. The cells were drug-treated for 24 hr and whole cell lysates were harvested and analyzed by Western blots. For detection of apoptotic markers, rabbit anti-Cleaved PARP (5625, Cell Signaling), 1:1000; rabbit anti-Cleaved Caspase-3 (9661, Cell Signaling) 1:1000 were used.

### mRNA extraction and analysis

Total RNA was extracted using TRIzol (Fisher Scientific). Superscript III and oligo(dT) (Fisher Scientific) was used to perform reverse transcription and cDNA was amplified using DNA Taq (NEB). RT-PCR was performed using the following primers: GRP78: Forward: 5′-CAGCACAGACAGATTGACCTAT-3′ and Reverse: p3: 5′-GACATCAGCACCGCACTTCTCA-3′; β*-*actin: Forward: 5′-TCGTGCGTGACATTAAGGAG-3′ and Reverse: 5′-AGCACTGTGTTGGCGTACAG-3′. *Xbp1*: Forward: 5′-CTGGAACAGCAAGTGGTAGA-3′ and Reverse: 5′-CTGGGTCCTTCTGGGTAGAC-3′. PCR products were resolved on a 2% agarose gel.

### Luciferase assay

The Luciferase Reporter Assay System (Promega) was used to perform the luciferase assay. For luciferase assay performed on HCT116, the cells were co-transfected with -169 luciferase (firefly) and Renilla luciferase expressing plasmids (Promega) for 4 hours and a dual luciferase assay was performed. Cells were then treated with either tunicamycin or thapsigargin alone or in combination with IT-139 for 6 hours before luciferase expression was tested following manufacturer guidelines. Firefly luciferase enzyme activity was normalized to the Renilla luciferase enzyme activity using the Dual Luciferase Reporter Assay System (Promega).

### Immunohistochemistry

Immunostaining of the paraffin-embedded tissue sections were performed using the antibody for GRP78 (1:400, Abcam #ab108613). Images were analyzed with ZEN lite imaging software (ZEISS).

### Cell viability assays

All viability assays were run in 96-wellpates at 72 hours. IC50 viability assays were determined by Cell-Titer Blue (Promega catalog # G8080) and Cell Titer-Glo Luminescent (Promega catalog # G7570). EC50 viability assay was evaluated using the MTT Cell Proliferation Assay Kit (ATCC catalog # 30–1010K).

### Animals

All experiments were carried out in 6–8 week old female nu/nu athymic mice (Charles River labs). The health of all animals was monitored daily by gross observation and analysis of blood samples of sentinel animals. All animals were allowed to acclimatize and recover from any shipping-related stress for a minimum of 72 h before experimental use. Autoclaved water and irradiated food were provided *ad libitum*, and the animals were maintained on a 12-h light and dark cycle. Cages, bedding and water bottles were autoclaved before use and were changed weekly. Prior to studies animals were inserted by subcutaneous injection of a radiofrequency identification transponder (RFIDs). Animals were weighed at study start and following treatment, three times a week. All animal experiments were done in accordance with protocols approved by the Institutional Animal Care and Use Committee. All animal treatments were administered intravenously via the lateral tail-vein, except for 5-FU administered intraperitoneal.

### Reagents

Oxaliplatin was purchased from Toronto Research Chemical lot#8-YM-26-1, 98% purity. Dissolution of oxaliplatin was in 150 mM saline at a concentration of 2 mg/mL. Cisplatin was purchased from TRC lot#12-ABY-28-1, 98% purity. Dissolution of cisplatin was in 150 mM saline at a concentration of 2 mg/mL. 5-Fluorouracil was obtained from American Pharmaceutical Partners, Inc. Dissolution was in 150 mM saline at a concentration of 10 mg/mL. IT-139 is lot TV-2-149, with a purity of 98% by HPLC. Dissolution of IT-139 was in 150 mM saline at a concentration of 10 mg/mL.

### Statistical analysis

Statistical analyses were performed using GraphPad Prism (GraphPad Software, Inc., La Jolla, CA USA). Plotted values for *in vivo* studies represent means ± SEM. A student’s *t* test was performed to assess statistical significance. *P* values of ≤ 0.05 designated ^*^P values of ≤ 0.01 designated as ^**^ and *P* values of ≤ 0.001 designated as ^***^ were considered statistically significant.

## SUPPLEMENTARY MATERIALS FIGURES


