# Supplementary material for: Suppression of stress induction of the 78-kilodalton glucose regulated protein (GRP78) in cancer by IT-139, an anti-tumor ruthenium small molecule inhibitor
Source: Oncotarget. 2018 Jul 3;9(51):29698–714. doi: 10.18632/oncotarget.25679 (PMC6049868; doi:10.18632/oncotarget.25679)
Supplement: Supplementary file 1 [file oncotarget-09-29698-s001.pdf]

Suppression of stress induction of the 78-kilodalton glucose regulated protein (GRP78) in cancer by IT-139, an anti-tumor ruthenium small molecule inhibitor

SUPPLEMENTARY MATERIALS

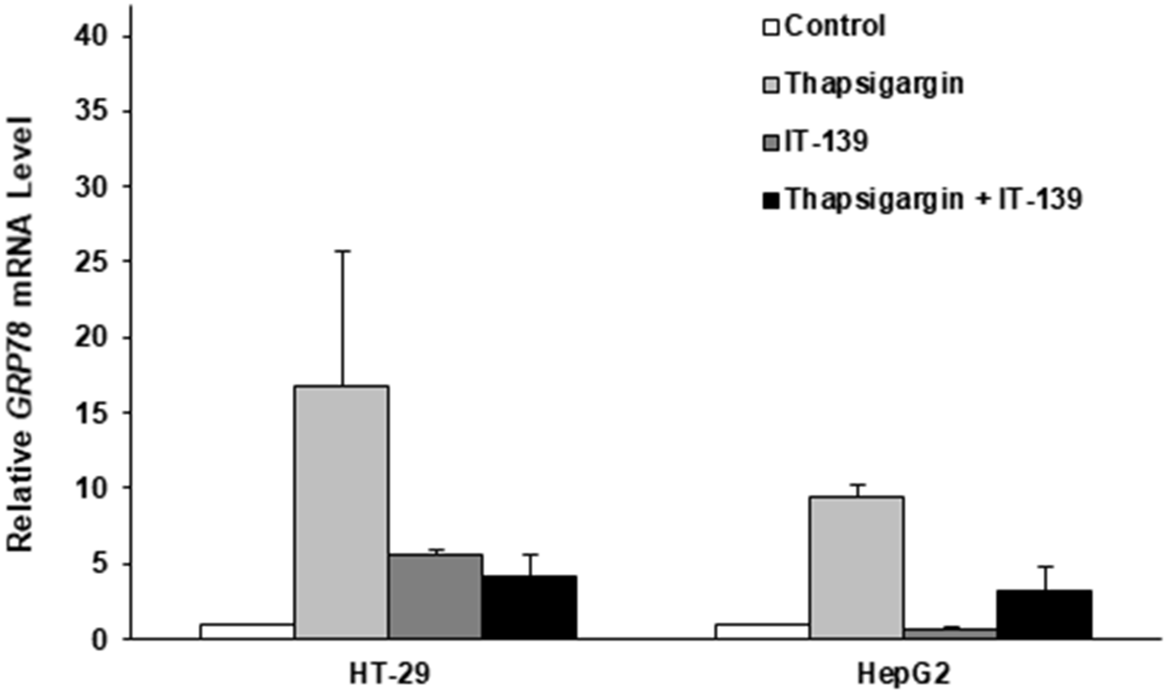

Supplementary Figure 1: IT-139 suppresses *GRP78* expression at the transcriptional level. The indicated cancer cell lines were treated as indicated and the *GRP78* mRNA levels were measured and plotted.

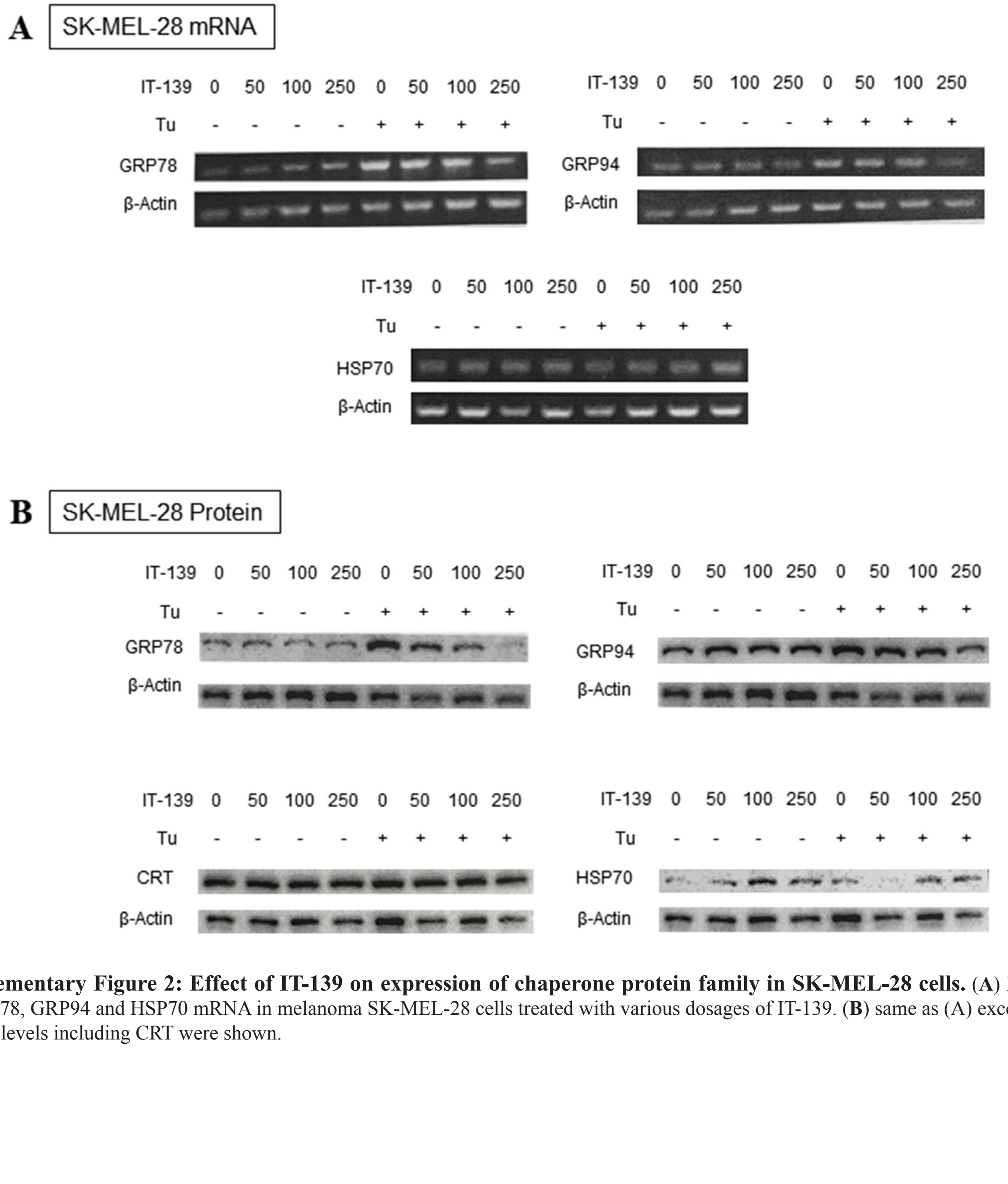

Supplementary Figure 2: Effect of IT-139 on expression of chaperone protein family in SK-MEL-28 cells. (**A**) Levels of GRP78, GRP94 and HSP70 mRNA in melanoma SK-MEL-28 cells treated with various dosages of IT-139. (**B**) same as (A) except the protein levels including CRT were shown.

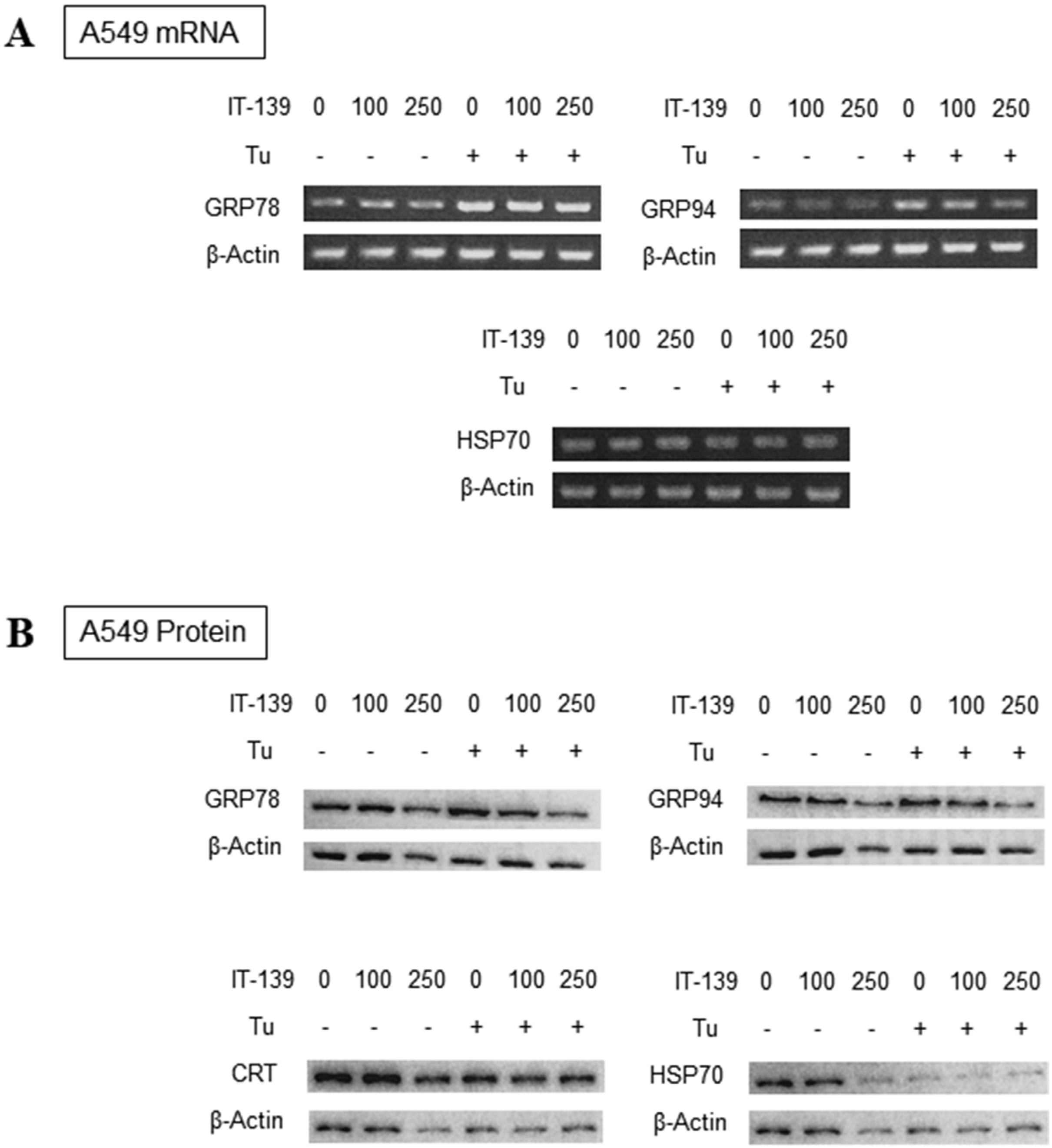

Supplementary Figure 3. Effect of IT-139 on expression of chaperone protein family in A549 cells. (**A**) Levels of GRP78, GRP94 and HSP70 mRNA in human lung cancer A549 cells treated with various dosages of IT-139. (**B**) same as (A) except the protein levels including CRT were shown.
